# The Potential Mitigating Effect of Curcumin Root Extract Against Synthetic Anabolic Steroid Boldenone-Induced Kidney Injury in Male Rats

**DOI:** 10.12688/f1000research.174653.2

**Published:** 2026-07-15

**Authors:** Asmaa M. Neamah, Sura M. Alkadhimy, Ruwaidah A.R. Abbas, Mohammed Y.I. Al-Hamadani, Mokhtar Ibrahim Yousef

**Affiliations:** 1 Department of Environment, College of Science, University of Al-Qadisiyah, Al Diwaniyah, Iraq; 2 Department of Biology, College of Science, University of Karbala, Karbala, Iraq; 3 Department of Pathological Analysis, College of Applied Sciences, University of Fallujah, Al-Fallujah, Iraq; 4 Department of Biology, College of Education, University of Fallujah, Al-Fallujah, Iraq; 5 Department of Environmental Studies, Institute of Graduate Studies and Research, Alexandria University, Alexandria, Egypt

**Keywords:** Anabolic-androgenic steroids, Boldenone, Curcumin root extract, Kidney injury, Oxidative stress, TNFα expressions, Nephroprotection, Male Wistar rats, Renal toxicity, Antioxidant therapy, Steroid-induced nephrotoxicity.

## Abstract

**Background:**

The anabolic-androgenic steroid boldenone is widely abused and is strongly linked to nephrotoxicity, primarily mediated through oxidative stress and inflammation. Curcumin, a natural polyphenol, is renowned for its potent antioxidant and anti-inflammatory properties, but its protective role against boldenone-induced renal injury is not fully elucidated. This study aimed to investigate the potential nephroprotective effects of curcumin against boldenone-induced kidney damage in a rat model, focusing on biochemical parameters, oxidative stress markers, inflammatory response, and histological changes.

**Methods:**

Thirty-two male Wistar rats were divided into four groups (n=8): control, curcumin-only (300 mg/kg/day), boldenone-only (5 mg/kg/week), and boldenone + curcumin. Over the experimental period, body weight, kidney weight, food/water intake, and serum renal function markers (urea, creatinine, electrolytes) were monitored. Renal tissue was analyzed for oxidative stress markers (MDA, GSH, CAT, SOD), the inflammatory cytokine TNF-α, and histological alterations.

**Results:**

Boldenone administration significantly increased body and kidney weights, serum urea, creatinine, potassium, and chloride, while decreasing sodium and calcium. It induced marked oxidative stress (elevated MDA, depleted GSH, CAT, SOD) and inflammation (elevated TNF-α), resulting in significant tubular necrosis and hypertrophy. Post-treatment with curcumin substantially mitigated these abnormalities, normalizing serum markers, restoring antioxidant defenses and electrolyte balance, reducing TNF-α expression, and ameliorating histological damage.

**Conclusion:**

Curcumin exerts significant nephroprotection against boldenone-induced toxicity through its antioxidant, anti-inflammatory, and cytoprotective mechanisms. These findings justify its exploration as a potential adjunct therapy to prevent steroid-associated kidney injury. Future studies should focus on elucidating the precise molecular signaling pathways involved and validating these effects in clinical scenarios of AAS abuse.

## Introduction

Anabolic-androgenic steroids (AASs) are artificial analogs of testosterone that increase its anabolic effects and reduce androgenic properties ( Fadah et al., 2023; Hussain et al., 2024). Their ability to stimulate muscle protein synthesis and increase lean body mass has enabled them to gain popularity among athletes who are interested in performance enhancement. In addition to sports, AASs have also found applications in veterinary medicine to stimulate muscle development in animals (racehorses and working dogs) ( Borodi et al., 2020; Islam et al., 2022; Ayubi et al., 2023).

Boldenone is one of these substances widely used as a growth promoter in animal rearing to increase weight gain and lower breeding expenses, which has questioned the ethical and safety issues about food quality and human exposure ( El-Moghazy et al., 2012; Tousson et al., 2012, 2016; Getabalew et al., 2020). Despite the boldenone being a Schedule III controlled substance rated to be used in veterinary practice, it has been used by athletes and bodybuilders illegally to build muscle. This abuse results in high systemic boldenone concentrations, which are frequently irreversible or irreparable organ damage especially nephrotoxicity.

As the abuse of AASs is increasingly becoming a widespread issue, the need to find natural compounds and other drugs with the ability to overcome their negative effects is on the rise ( Abd El-Zaher et al., 2025; Abosharaf et al., 2025). Phytochemicals possessing strong antioxidant and anti-inflammatory activities have been of particular interest as to their therapeutic purpose ( Essawy et al., 2022; Shalaby et al., 2023; Hasan et al., 2024). Curcumin is one of such compounds that are polyphenolic pigments of Curcuma longa (turmeric) which have extensive biological effects, such as anti-inflammatory, antioxidant, antimicrobial, and anticancer impacts ( Wang et al., 2012; Alotaibi et al., 2021; Islam et al., 2022; Radwan et al., 2024).

The cytoprotective and antioxidant properties of curcumin involve the scavenging of reactive oxygen species (ROS), the control of the inflammatory response, and the stimulation of home antioxidant defense mechanisms ( Samarghandian et al., 2017; Damiano et al., 2020). These attributes indicate that curcumin is possible to relieve boldenone-induced kidney oxidative stress and damage.

Thus, the hypothesis of the present research is that curcumin root extract will help to prevent oxidative stress and kidney damage as caused by boldenone in male rats. It is aimed at testing the possible nephroprotective effect of curcumin and giving information about the possible ways of its use in the treatment of anabolic steroid-induced nephrotoxicity.

Nephrotoxicity through multiple interconnected mechanisms, including excessive generation of reactive oxygen species (ROS), lipid peroxidation, and activation of pro-inflammatory pathways such as TNF-α signaling ( Dornelles et al., 2017; Frankenfeld et al., 2014). In addition, these compounds may disrupt renal electrolyte homeostasis through interference with membrane transport systems, including Na ^+^/K ^+^-ATPase, as suggested in previous studies ( Tousson et al., 2018; Elmasry et al., 2018). Structural alterations such as glomerular hyperfiltration and tubular hypertrophy further contribute to progressive renal injury ( Sessa et al., 2020). Clinically, these pathological changes are reflected in elevated serum creatinine and urea levels, along with electrolyte disturbances, which are widely recognized as systemic biomarkers of steroid-induced nephrotoxicity ( El-Moghazy et al., 2012; Tousson et al., 2016).

## Materials and methods

### Animal model and housing

Thirty-two healthy male Wistar albino rats (180–200 g, 12–16 weeks old) were obtained from the Drug Control Center in Baghdad, Iraq. Animals were acclimatized for two weeks under controlled laboratory conditions (22 ± 2°C, 50–70% relative humidity, and a 12-hour light/dark cycle). During acclimation, rats were provided with a standard laboratory diet and water *ad libitum.*

All procedures were performed in accordance with the International Council for Laboratory Animal Science (ICLAS) Guide for the Care and Use of Laboratory Animals and the ARRIVE guidelines. The study protocol was reviewed and approved by the Institutional Animal Care and Use Committee (IACUC) of Fallujah University, College of Applied Sciences (Ethical approval no. UOF.CAS.06-240311).

***Experimental design***

Rats were randomly assigned to four equal groups (n = 8):

• **Group 1 (Control):** Received no treatment.
• **Group 2 (Curcumin):** Administered curcumin orally (300 mg/kg body weight/day for 2 weeks) following Radwan et al. (2023).
• **Group 3 (Boldenone):** Received intramuscular injections of boldenone (5 mg/kg body weight/week for 4 weeks) according to Alm-Eldeen and Tousson (2012).
• **Group 4 (Boldenone + Curcumin):** Exposed to boldenone for 4 weeks, followed by 2 weeks of oral curcumin supplementation (300 mg/kg/day).

The administered preparation consisted of a curcuminoid-rich Curcuma longa rhizome extract obtained in powder form and freshly suspended in distilled water prior to administration. The selected dose (300 mg/kg/day) was chosen based on previously published nephroprotection studies. The preparation represents a standardized extract commonly used in experimental pharmacological studies to ensure reproducibility of biological activity.

Throughout the experiment, body weight, food intake, and water consumption were recorded weekly. The following formula was used to get the relative kidney weight (RKW):

$$\mathrm{RKW}=\frac{\text{Kidney weight}}{\text{Body weight}}\times 100$$

***Blood and tissue collection***

After overnight fasting (10–12 h), rats were anesthetized with pentobarbital and euthanized. To ensure total sedation of the rats before euthanasia, all rats were anesthetized by injecting them with pentobarbital at a concentration of 50 mg/kg body weight into the peritoneal cavity. Loss of pedal reflex and loss of corneal reflex were used to confirm the depth of anesthesia. After anesthesia, animals were exsanguinated by cardiac puncture. Every practice was carried out with compliance with the American Veterinary Medical Association (AVMA) Guidelines to the Euthanasia of Animals (2020) and the Institutional Animal Care and Use Committee (IACUC) of Fallujah University, College of Applied Sciences (Ethical approval no. UOF.CAS.06-240311).

Blood samples were collected via the inferior vena cava into non-heparinized tubes, allowed to clot, and centrifuged at 860 × g for 20 min. Serum was separated and stored at –80°C for biochemical assays.

Kidneys were excised, washed in chilled 0.9% saline, and cleared of fat and connective tissue. A 10% (w/v) tissue homogenate was prepared using a Potter–Elvehjem homogenizer in ice-cold 0.01 M sodium phosphate buffer (pH 7.4) containing 1.15% KCl. Homogenates were centrifuged at 10,000 × g for 20 min at 4°C, and the supernatant was stored at –20°C for further biochemical analysis.

***Kidney function and electrolyte assessment***

Serum creatinine and urea were estimated according to Patton and Crouch (1977). Electrolytes (sodium, potassium, calcium, and chloride) were measured using commercial diagnostic kits (Sensa-core, India) as described by Abd Eldaim et al. (2019).

***Antioxidant and oxidative stress assays***

Renal oxidative stress and antioxidant parameters were determined as follows:

*Malondialdehyde (MDA):* Estimated as a marker of lipid peroxidation using a Biodiagnostic kit (Egypt) following Mesbah et al. (2004).

*Reduced Glutathione (GSH):* Measured by the method of Teare et al. (1993) using 5,5′-dithiobis-(2-nitrobenzoic acid) and expressed as μmol GSH/g tissue.

*Catalase (CAT):* Activity determined spectrophotometrically by monitoring the decomposition of H _2_O _2_ at 240 nm, following Saggu et al. (2014).

*Superoxide Dismutase (SOD):* Activity assessed using the inhibition of adrenaline auto-oxidation according to Misra and Fridovich (1972).

### Histopathological examination

Kidney specimens from different groups were isolated, fixed in 10% neutral buffered formalin, paraffin embedded, sectioned and stained with hematoxylin and eosin after Tousson (2016).

### Immuno-histochemical detection

Following Tousson et al. (2025) immuno-labeling of tumor necrosis factor alpha (TNFα) were 5 μm kidney sections onto positively charged slides. Distribution of TNFα stained cytoplasm were examined in deparaffinized sections using an Avidin–Biotin–Peroxidase immunohistochemical method (Elite–ABC, Vector Laboratories, CA, USA) with TNFα monoclonal antibody (dilution 1:100; DAKO Japan Co, Tokyo, Japan).

### Statistical analysis

Data are expressed as mean ± SE. Statistical comparisons were performed using one-way ANOVA followed by post hoc analysis. Symbols * indicate significant differences compared to the control group, whereas (#) indicate significant differences compared to the boldenone-treated group at *p* < 0.05.

## Results

Table 1 presents the effects of boldenone (ASB) administration and curcumin treatment on body weight, relative kidney weight, and feed and water consumption. Rats treated with boldenone showed a significant increase in body and relative kidney weights, as well as in feed and water intake, compared to the control and curcumin-only groups. However, post-treatment with curcumin (ASB + Cur) markedly ameliorated these alterations, demonstrating a normalizing effect and indicating that curcumin mitigates the physiological disturbances induced by boldenone.

**Table 1.** Effects of Curcumin and Boldenone, Alone and in Combination, on Physiological Parameters in Rats: Water Intake, Feed Intake, Relative Body Weight (RBW), and Relative Kidney Weight (RKW).

Parameter	Experimental groups			
	Control	Cur	ASB	ASB + Cur
**Water intake (ml/rat/day)**	27.4 ^**#**^ ± 1.68	29.2 ^**#**^ ± 1.55	42.4* ± 2.38	33.6 ^#^* ± 1.84
**Food intake (g/rat/day)**	12.6 ^**#**^ ± 0.71	12.4 ^**#**^ ± 0.84	19.4* ± 1.09	12.9 ^**#**^ ± 0.87
**RBW (g/100 g)**	27.0 ^**#**^ ± 1.41	25.3 ^**#**^ ± 1.45	44.0* ± 2.81	38.3 ^#^* ± 1.75
**RKW (g/100 g)**	2.46 ^**#**^ ± 0.16	2.39 ^**#**^ ± 0.16	4.14* ± 0.19	2.70 ^**#**^ ± 0.20

Data are expressed as mean ± SE. Statistical comparisons were performed using one-way ANOVA followed by post hoc analysis. Symbols (*) indicate significant differences compared to the control group, whereas (#) indicate significant differences compared to the boldenone-treated group at *p* < 0.05.

Table 2 shows a remarkable increase in serum creatinine and urea levels of the rat subjected to boldenone relative to the control and curcumin-treated rats indicating a failure of the kidneys to perform their functions. Conversely, post-treatment with curcumin after boldenone (ASB + Cur) substantially decreased both parameters substantially as compared to the ASB group implying a nephroprotective effect of curcumin on boldenone-induced kidney malfunction.

**Table 2.** Alterations in the serum urea and creatinine levels of rats given boldenone (ASB), curcumin (Cur.), or both.

Parameter	Experimental groups			
	Control	Cur	ASB	ASB+Cur
**Creatinine (mg/dl)**	0.61 ^**#**^ ± 0.05	0.59 ^**#**^ ± 0.03	0.98* ± 0.05	0.65 ^**#**^ ± 0.04
**Urea (mg/dl)**	23.0 ^**#**^ ± 0.90	24.5 ^**#**^ ± 0.89	37.2* ± 1.15	27.0 ^**#**^ ± 1.26

Data are expressed as mean ± SE. Statistical comparisons were performed using one-way ANOVA followed by post hoc analysis. Symbols (*) indicate significant differences compared to the control group, whereas (#) indicate significant differences compared to the boldenone-treated group at *p* < 0.05.

Table 3 shows the impact of boldenone and curcumin on serum levels of electrolytes. The administration of boldenone resulted in a significant reduction in sodium and calcium and a significant increase in potassium and chloride and a significant decrease in chloride levels, compared to the control and curcumin groups. On the other hand, ASB + Cur co-treatment leveled these electrolytes significantly, increasing the levels of sodium and calcium, decreasing those of potassium and chloride levels compared to the ASB. These results show that curcumin is a good solution to the electrolyte imbalances which boldenone causes.

**Table 3.** Changes in the levels of electrolytes of rats treated with curcumin (Cur.), boldenone (ASB), and their combination.

Parameter	Experimental groups			
	Control	Cur	ASB	ASB + Cur
**K** ^**+**^ **(mmol/l)**	3.85 ^**#**^ ± 0.16	3.65 ^**#**^ ± 0.21	4.35* ± 0.18	4.04* ^**#**^ ± 0.22
**Na** ^**+**^ **(mmol/l)**	138.5 ^**#**^ ± 5.42	139.9 ^**#**^ ± 4.41	131.0* ± 6.12	138. 5 ^**#**^ ± 5.25
**Ca** ^**++**^ **(mmol/l)**	1.22 ^**#**^ ± 0.10	1.21 ^**#**^ ± 0.09	1.16* ± 0.08	1.21 ^**#**^ ± 0.09
**Cl** ^**-**^ **(mmol/l)**	102.0 ^**#**^ ± 8.36	101.5 ^**#**^ ± 9.01	112.5* ± 8.55	102.0 ^**#**^ ± 9.45

Data are expressed as mean ± SE. Statistical comparisons were performed using one-way ANOVA followed by post hoc analysis. Symbols (*) indicate significant differences compared to the control group, whereas (#) indicate significant differences compared to the boldenone-treated group at *p* < 0.05.

According to Table 4, the boldenone treatment had a significant effect on the renal malondialdehyde (MDA) levels, significantly reduced the concentration of glutathione (GSH) and activity of antioxidant enzymes catalase (CAT) and superoxide dismutase (SOD) in comparison with the control and curcumin groups. Nevertheless, the co-treatment with curcumin (ASB + Cur) had a significant effect on decreasing the lipid peroxidation rates by lowering the level of MDA and, at the same time, returning the GSH, CAT, and SOD levels to almost normal levels in comparison with the ASB group. These findings validate the assumptions that curcumin has strong antioxidant and cytoprotective effects in boldenone-induced oxidative renal injury.

**Table 4.** Changes in enzymatic and non-enzymatic antioxidant parameters of rats treated with curcumin (Cur.), boldenone (ASB), and their combination.

Parameter	Experimental groups			
	Control	Cur	ASB	ASB+Cur
**MDA (nmole/g tissue)**	30.5 ^#^ ± 1.25	26.8 ^#^ ± 1.50	60.5* ± 2.75	33.4 ^#^ ± 1.31
**GSH (mmol/mg protein)**	3.25 ^#^ ± 0.11	3.38 ^#^ ± 0.09	1.18* ± 0.11	3.12 ^#^ ± 0.12
**CAT (U/mg protein)**	36.3 ^#^ ± 1.45	42.5 ^#^ ± 2.91	18.5* ± 1.04	33.5 ^#^ ± 1.33
**SOD (U/mg protein)**	40.0 ^#^ ± 2.40	44.8 ^#^ ± 2.35	20.5* ± 1.45	38.5 ^#^ ± 2.16

Data are expressed as mean ± SE. Statistical comparisons were performed using one-way ANOVA followed by post hoc analysis. Symbols (*) indicate significant differences compared to the control group, whereas (#) indicate significant differences compared to the boldenone-treated group at *p* < 0.05.

### Impact of Cur on histopathological examination

Normal architecture with normal renal tubules and glomeruli were detected in kidney sections in both control and Cur groups ( Figure 1A & 1B). Conversely, kidney sections in treated rats with ASB revealed severe hypertrophy in tubular cells and marked necrotic tubular cells ( Figure 1C). Treated rats with Cur after ASB treatments (ASB+Cur) showed a good degree of kidney improvements with only mild hypertrophy in glomeruli and tubular cells ( Figure 1D).

**Figure 1.**
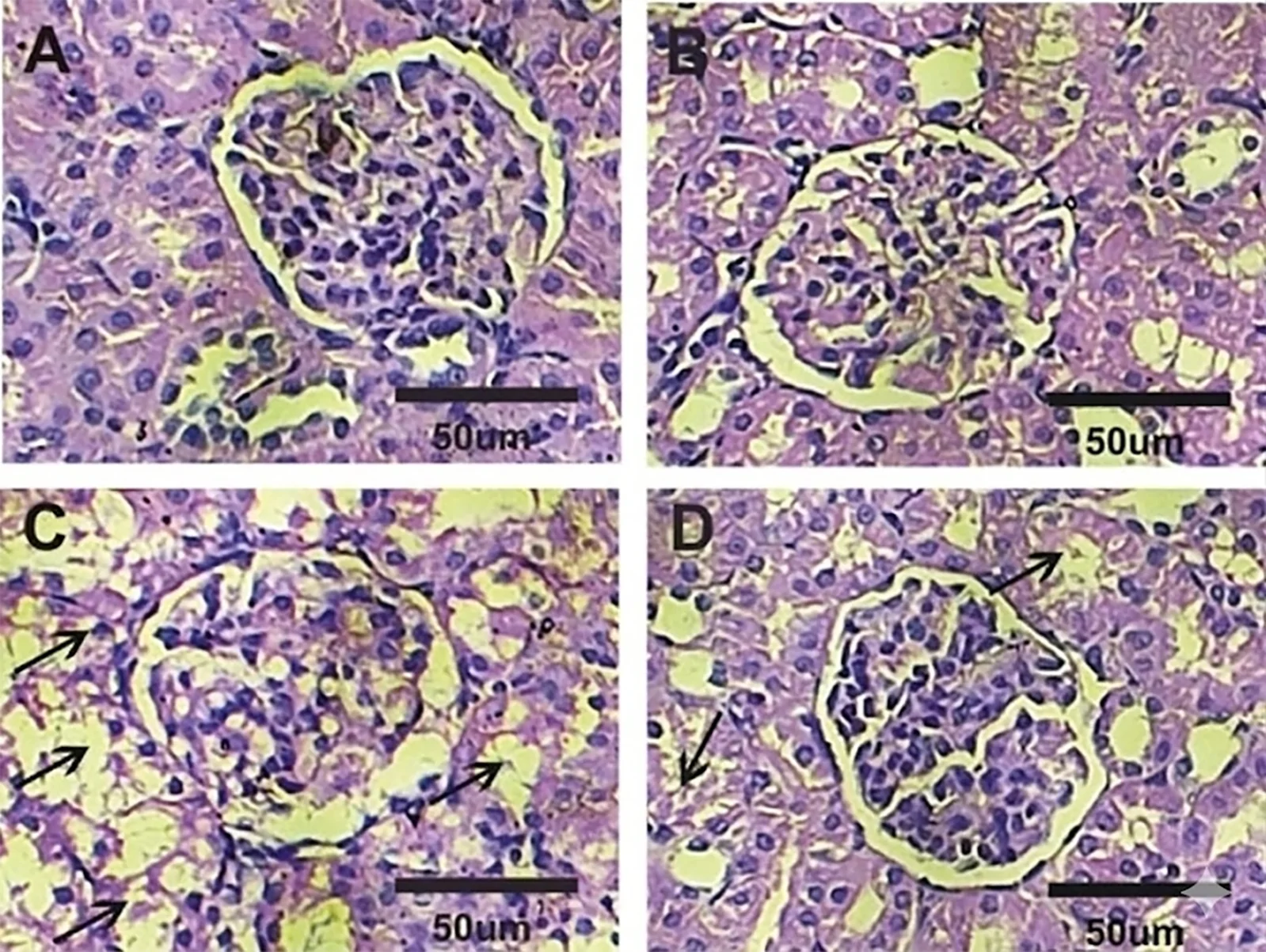
Photo-micrographs of H&E -stained kidney sections. (A–B) Control and curcumin-treated groups showed normal renal architecture with intact glomeruli and well-organized renal tubules, without signs of degeneration or inflammatory infiltration. (C) Boldenone-treated group showing severe histopathological alterations, including tubular epithelial degeneration, cellular necrosis, and marked structural disorganization. (D) Post-treatment with curcumin showed significant improvement in renal histology, with restoration of normal tubular architecture and only mild residual degenerative changes.

### Impact of Cur on TNFα expressions

Figure 2 revealed the changes in TNFα expressions in kidney sections in different groups. Negative or faint positive reactions were detected in control and Cur groups while a moderate positive reaction was detected in ASB group ( Figure 2A- 2C). On the other hand; treated rats with Cur after ASB treatments (ASB+Cur) showed mild positive reaction for TNFα ( Figure 2D).

**Figure 2.**
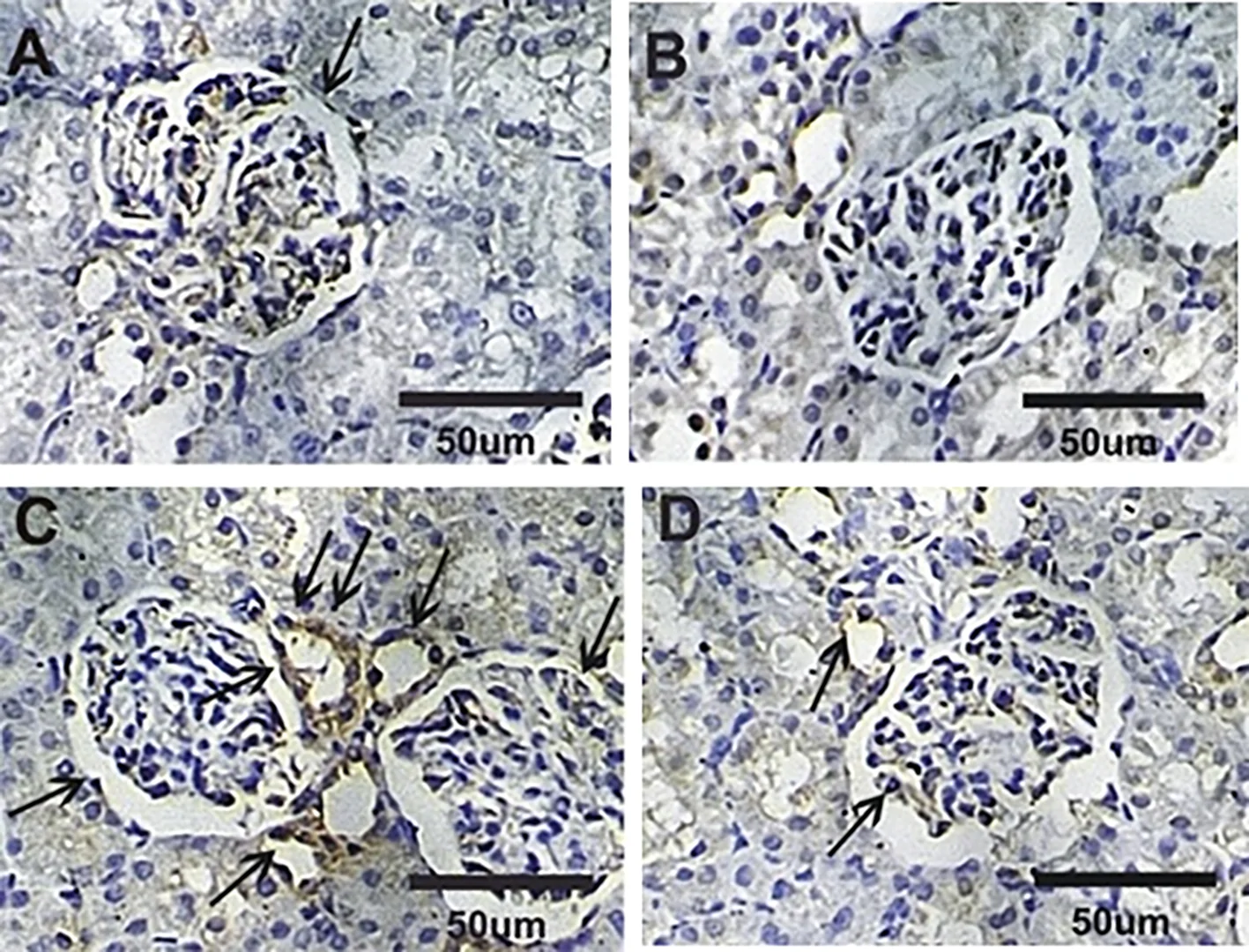
Photomicrographs of TNF-α immunoexpression in kidney sections. (A–B) Control and curcumin-treated groups showed negative to faint cytoplasmic TNF-α expression. (C) The Boldenone-treated group showed moderate positive TNF-α immunoreactivity, indicating enhanced inflammatory response. (D) Post-treatment with curcumin showed reduced TNF-α expression, reflecting attenuation of inflammation.

## Discussion

The increase in body weight gain observed following boldenone administration in this study aligns with the findings of El-Moghazy et al. (2012), who reported similar effects in male rabbits. This growth was coupled with increasing feed and water consumption and relative kidney weight, which is in agreement with the present findings. Equally, Hoseini et al. (2009) resulted that the body and kidney weights of mice in the study had risen due to the administration of nandrolone decanoate. Conversely, Chang et al. (2021) also established a body and kidney weight increase reduction of curcumin root extract over the control, which confirms our observation of mitigating effect of curcumin. Moreover, Ali et al. (2018) revealed that the treatment with curcumin reduced the relative kidney weight, water consumption, urine production, and weight gain, which additionally confirmed our data.

The changes in serum calcium, sodium, creatinine, urea, potassium and chloride in the current study all point to the fact that boldenone undecylenate growth promoter abuses cause structural and functional impairment of the kidney, which may cause secondary and progressive kidney failure. These results are in agreement with El-Moghazy et al. (2012) and Tousson et al. (2016) studies which found that the use of boldenone led to a marked increase in serum urea and creatinine concentration in rabbits, and that boldenone was indeed nephrotoxic.

In a similar study, Alm-Eldeen and Tousson (2012) showed that the injection of boldenone had significant effect on increasing the serum total protein, urea, and creatinine level and reducing the albumin/globulin (A/G) ratio in male rabbits. These observations are supported by our results and are consistent with the study conducted by Tousson et al. (2016), who demonstrated that hepatic and renal toxicity were severe following the administration of boldenone.

Mechanistically, high concentrations of anabolic steroid have been reported to suppress the Na ^+^/K ^+^-ATPase activity thus causing calcium and sodium ion to build up in the cell, hence interference with the electrolyte homeostasis. The electrolyte imbalances reported in this study are in line with those reported by Tousson et al. (2018) and Elmasry et al. (2018). There are even suggestions that AASs may be able to damage renal glomeruli directly through the induction of glomerular hyperfiltration and hypertrophy ( Sessa et al., 2020).

The protective effect of curcumin that was realized in this study is in line with what has been reported before. Venkatesan et al. (2000) also showed that curcumin prevented adriamycin-induced nephrotoxicity in rats and Ueki et al. (2013) established that curcumin inhibited renal inflammation and cisplatin-induced nephrotoxicity in mice. Equally, Damiano et al. (2020) stated that curcumin rescued rat kidneys exposed to ochratoxin-induced injury, which also indicates the broad renoprotective nature of curcumin.

The results of this study on the oxidative stress are also consistent with the results reported by earlier researchers that anabolic steroids cause redox imbalance and lipid peroxidation. Dornelles et al. (2017) showed that steroid exposure was associated with heightened hepatic and renal oxidative stress, and Frankenfeld et al. (2014) also described multi-organ damage (liver, heart, kidneys) caused by nandrolone decanoate treatment. The renal toxicity here also favors the nephrotoxic effects of Tsitsimpikou et al. (2016) and cardiac oxidative stress of Kara et al. (2018) after stanozolol use.

The high antioxidant activity exhibited by Curcumin in this case as indicated by the increase of GSH, CAT, and SOD activity and decrease of MDA levels prove that this compound can prevent lipid peroxidation and oxidative stress. It is in line with Tokaç et al. (2013) who emphasized the curcumin action of protecting renal tissue against oxidative and DNA damage. Equally, Samarghandian et al. (2017) and Ali et al. (2018) found similar renoprotective effects in chronic kidney disease and oxidative stress models. Together, these findings support the idea that the curcumin has a potent nephroprotective impact in terms of antioxidant, anti-inflammatory, and cytoprotective properties.

The current paper proves the protective action of Cur against the ASB-induced renal damage through the histopathological changes and the TNF changes in the expression. The ASB exposure led to significant distortion of normal renal architecture with severe tubular hypertrophy and massive necrotic alterations. The results are corroborated by the existing literature that shows that ASB as well as other analogous chemical stressors cause oxidative stress and inflammation, which eventually result in tubular damage and cellular loss of integrity. This is also in accordance with Tousson et al. (2025) who concludes that; nandrolone decanote as steroids caused renal toxicity and inflammation in male rats.

The observed electrolyte imbalance may be partially explained by mechanisms reported in previous studies ( Tousson et al., 2018; Elmasry et al., 2018), including potential impairment of Na ^+^/K ^+^-ATPase activity; however, this parameter was not directly assessed in the present study and should therefore be interpreted cautiously.

## Conclusion

In conclusion, boldenone administration induces significant renal dysfunction associated with oxidative stress, inflammation, and histopathological alterations in male rats. Curcumin root extract demonstrated a protective effect by ameliorating these changes, likely through its antioxidant and anti-inflammatory properties. However, these findings are limited to an experimental animal model, and further studies are required to elucidate the underlying molecular mechanisms and to confirm their relevance in clinical settings.

## Consent to participate

Not applicable.

## Declaration of generative AI Use in the writing process

In the course of drafting this manuscript, the authors engaged the generative AI tool Perplexity during the middle editorial stage to help with the phrasing, and we revisited the text to improve the clarity, fluency, and readability to the text. After AI-assisted revisions, authors reviewed, changed, and sanctioned all the texts to make sure they were correct and complete. Responsibility for the text lies with the author, and the use of AI has not affected the scientific quality and conclusions of the work.

## Data Availability

The datasets supporting the conclusions of this study, including the completed ARRIVE 2.0 checklist and raw experimental data, are publicly available in the Zenodo repository. Repository: Zenodo Title: ARRIVE Checklist and Supporting Data for The Potential Mitigating Effect of Curcumin Root Extract Against Synthetic Anabolic Steroid Boldenone-Induced Kidney Injury in Male Rats DOI: https://doi.org/10.5281/zenodo.18133897 Neamah et al. (2026) License: Creative Commons Zero (CC0 1.0 Public Domain Dedication) The dataset includes:
• ARRIVE 2.0 Author Checklist (PDF) • Raw experimental data files (Excel and PDF) All data are freely accessible without restriction.
