# Supplementary material for: The Potential Mitigating Effect of Curcumin Root Extract Against Synthetic Anabolic Steroid Boldenone-Induced Kidney Injury in Male Rats
Source: F1000Res. 2026 Aug 29;15:64. Originally published 2026 Jan 16. [Version 3] doi: 10.12688/f1000research.174653.3 (PMC13731895; doi:10.12688/f1000research.174653.3)
Supplement: Peer review history

# Reviewer report: Reviewer response for version 3

**DOI:** 10.5256/f1000research.208691.r515919

**Published:** 2026-09-18

**Authors:** Daniela Joyce Trujillo

**Affiliations:** 1 Instituto Potosino de Investigación Científica y Tecnológica (DMA-IPICYT), San Luis, Mexico

The authors have satisfactorily resolved most of the major concerns raised during the previous review period, and the revised manuscript has significantly improved the clarity, methodological description, and interpretation of the results. In particular, clarification of the post-treatment design, moderation of discussions on the activity of Na+/K+-ATPase, and revision of the histological presentation strengthened the manuscript. However, there are still some minor problems. First, since curcumin was administered after exposure to bartonone as a post-treatment intervention, the term “protective effect” in the conclusion should preferably be replaced by  “therapeutic effect” or a similar term in order to maintain consistency with the experimental design. Secondly, the specific post-hoc test used after a single ANOVA must be explicitly stated in the methods and/or table legends rather than only referring to “post-hoc analysis”. Thirdly, although the authors have indicated in their responses that statistical clarifications have been added to the legends of Tables 1–4, this information is not consistently incorporated into the footnotes of the revised table. The relevant table legends must clearly indicate the format of data presentation, the statistical test used and the specific post-hoc test, the significance threshold, and the meaning of all symbols showing statistical differences. Once these minor points have been resolved, I consider the manuscript to be suitable for indexing.

Is the work clearly and accurately presented and does it cite the current literature?

Partly

If applicable, is the statistical analysis and its interpretation appropriate?

Partly

Are all the source data underlying the results available to ensure full reproducibility?

Partly

Is the study design appropriate and is the work technically sound?

Partly

Are the conclusions drawn adequately supported by the results?

Partly

Are sufficient details of methods and analysis provided to allow replication by others?

Partly

Reviewer Expertise:

Physiology renan and cardiometabolic

I confirm that I have read this submission and believe that I have an appropriate level of expertise to confirm that it is of an acceptable scientific standard, however I have significant reservations, as outlined above.

# Response

**Published:** 2026-10-18

**Authors:** Mohammed AlHamadani

**Affiliations:** Department of Biology, College, University of Fallujah, Al-Fallujah, Al Anbar Governorate, Iraq

**Dear Reviewer (Daniela Joyce Trujillo),** Thank you very much for your thoughtful and constructive evaluation of our revised manuscript (Version 3), and for noting the improvements made. We sincerely appreciate your valuable guidance. We have fully addressed all the minor points you raised to prepare the manuscript for Version 4:

1. **Terminology in Conclusion:** We have revised the term "protective effect" to "therapeutic effect" (and "mitigating effect") in both the Abstract and Conclusion sections to align accurately with our post-treatment experimental design.
2. **Specific Post-Hoc Test:** We have explicitly stated the specific post-hoc test used ( **Tukey’s post-hoc test**) in the Materials and Methods section as well as in all table legends, rather than using general terms.
3. **Table Legends and Footnotes (Tables 1–4):** We have thoroughly updated and standardized the footnotes for Tables 1–4. Each legend now explicitly details the format of data presentation (Mean± SE), the statistical tests performed (One-way ANOVA followed by **Tukey’s post-hoc test**), the significance threshold ( *P* < 0.05), and a clear explanation of all superscript letters indicating statistical differences.

Thank you again for your time and for helping us enhance the quality and rigour of our manuscript for indexing. Best regards, **Mohammed Y.I. Al-Hamadani** *Corresponding Author*

# Reviewer report: Reviewer response for version 3

**DOI:** 10.5256/f1000research.208691.r515918

**Published:** 2026-09-12

**Authors:** Dawei Jiang

**Affiliations:** 1 Huazhong University of Science and Technology, Wuhan, Hubei, China

I have no further questions.

Is the work clearly and accurately presented and does it cite the current literature?

Yes

If applicable, is the statistical analysis and its interpretation appropriate?

Partly

Are all the source data underlying the results available to ensure full reproducibility?

Partly

Is the study design appropriate and is the work technically sound?

Partly

Are the conclusions drawn adequately supported by the results?

Partly

Are sufficient details of methods and analysis provided to allow replication by others?

Partly

Reviewer Expertise:

NA

I confirm that I have read this submission and believe that I have an appropriate level of expertise to confirm that it is of an acceptable scientific standard.

# Reviewer report: Reviewer response for version 2

**DOI:** 10.5256/f1000research.203920.r501798

**Published:** 2026-08-04

**Authors:** Dawei Jiang

**Affiliations:** 1 Huazhong University of Science and Technology, Wuhan, Hubei, China

The authors have addressed several presentation-related concerns, but important methodological and interpretational issues remain unresolved

1. The authors now describe curcumin administration as a post-treatment intervention, but the groups appear to have different total experimental durations. The boldenone group receives treatment for four weeks, whereas the boldenone-plus-curcumin group receives four weeks of boldenone followed by two additional weeks of curcumin. It is unclear whether the boldenone group received vehicle during these additional two weeks and whether all control groups were maintained for six weeks.
2. No renal measurements are reported immediately after the four-week boldenone exposure and before curcumin administration.
3. The statement that the material was a “standardized curcuminoid-rich extract commonly used in experimental pharmacological studies” does not provide adequate reproducibility. The authors should identify the supplier, batch number, extraction method, curcuminoid composition, purity or percentage of curcumin, storage conditions, and analytical characterization. The manuscript should also consistently distinguish between crude *Curcuma longa* extract and purified curcumin.
4. In the Results section, chloride is described as both increased and decreased after boldenone administration, although Table 3 shows an increase from 102.0 to 112.5 mmol/L. This must be corrected. In addition, the Results section still refers to the ASB+Cur group as receiving “co-treatment,” despite the revised designation as post-treatment. All remaining instances of “co-treatment,” “prevention,” and “simultaneous administration” should be systematically checked and corrected.

**Minor Comments**

- The dose rationale should specify whether 300 mg/kg/day refers to the total extract mass or the calculated curcumin content.
- The route and formulation of boldenone should be described more precisely, including the commercial preparation and vehicle.
- Figure 1 still displays a pronounced yellow-green background in the H&E images. The authors should provide raw or minimally processed images and state how brightness, contrast, and color balance were adjusted.
- Figure legends should indicate whether the images are representative of all animals and should include the number of animals analyzed.

Is the work clearly and accurately presented and does it cite the current literature?

Yes

If applicable, is the statistical analysis and its interpretation appropriate?

Partly

Are all the source data underlying the results available to ensure full reproducibility?

Partly

Is the study design appropriate and is the work technically sound?

Partly

Are the conclusions drawn adequately supported by the results?

Partly

Are sufficient details of methods and analysis provided to allow replication by others?

Partly

Reviewer Expertise:

Drug development for organ injury, in vivo drug delivery, nanomedicine, biomedical engineering, radiopharmaceuticals, molecular imaging

I confirm that I have read this submission and believe that I have an appropriate level of expertise to confirm that it is of an acceptable scientific standard, however I have significant reservations, as outlined above.

# Response

**Published:** 2026-08-15

**Authors:** Mohammed AlHamadani

**Affiliations:** Department of Biology, College, University of Fallujah, Al-Fallujah, Al Anbar Governorate, Iraq

Dear Editors of F1000Research,   We are pleased to submit the revised version of our manuscript entitled “The Potential Mitigating Effect of Curcumin Root Extract Against Synthetic Anabolic Steroid Boldenone-Induced Kidney Injury in Male Rats.”   We sincerely thank the Editorial Team and Reviewer 2 (Dr. Dawei Jiang) for the careful reassessment and constructive comments. We have undertaken a comprehensive revision focusing on experimental timeline, methodological transparency, reproducibility, data consistency, histological presentation, and interpretation.   The major revisions include clarification of the sequential post-treatment design; correction of the chloride description to match Table 3; systematic removal of inconsistent co-treatment/simultaneous-administration terminology; explicit acknowledgment of the absence of an intermediate renal measurement; further clarification of the Curcuma longa extract used and the distinction between the extract and purified curcumin; and revision of the histological figure legends and methodological descriptions.   All substantive changes made in response to Reviewer 2 are highlighted in red in the revised manuscript. A detailed point-by-point response is provided below.   Sincerely, Mohammed Y.I. Al-Hamadani On behalf of all authors   --------------------------------------------------------------------------------   Response to Reviewer 2 – Dawei Jiang   All revisions made in the manuscript in response to Reviewer 2 are highlighted in red in the revised manuscript for ease of identification and review.   We sincerely thank the reviewer for the careful and critical reassessment of our manuscript and for the constructive comments concerning experimental design, methodological reproducibility, data presentation, and interpretation. We have carefully considered all comments and revised the manuscript accordingly.  

 Comment 1 – Experimental timeline and post-treatment design Response: We thank the reviewer for highlighting this important issue. We confirm that Group 4 received boldenone for 4 weeks followed by oral Curcuma longa extract administration for 2 weeks; therefore, curcumin was administered sequentially after the boldenone exposure period and not concurrently with boldenone. The terminology has been standardized throughout the manuscript to “post-treatment”, and references to “co-treatment” and “simultaneous administration” have been removed where inconsistent with the experimental sequence. The corresponding experimental-sequence clarification has been added to the Materials and Methods section and the relevant Results and table descriptions have been revised.  

 Comment 2 – Intermediate renal measurements Response: We appreciate this important observation. No separate renal biochemical measurements were performed immediately after the 4-week boldenone exposure and before initiation of curcumin administration. We have therefore moderated the interpretation and explicitly acknowledged this limitation. The revised Discussion states that the study does not permit direct assessment of the temporal progression of renal injury or quantitative comparison of renal status immediately before and after curcumin intervention, and that future studies should incorporate serial measurements.  

 Comment 3 – Curcumin extract characterization and reproducibility Response: We thank the reviewer for emphasizing the importance of reproducibility. The Materials and Methods section has been revised to consistently identify the administered material as a Curcuma longa rhizome extract in powder form, administered orally at 300 mg/kg body weight/day, and to distinguish the extract preparation from purified curcumin. We will only include supplier, batch number, extraction method, purity/composition, storage, and analytical-characterization details that are verifiable from the original experimental records or product documentation; no unsupported information has been inferred.  

 Comment 4 – Chloride discrepancy and residual terminology Response: We sincerely thank the reviewer for identifying the inconsistency. The Results text has been corrected so that chloride is described as increased in the boldenone-treated group, consistent with Table 3 (102.0 ± 8.36 mmol/L in the control group versus 112.5 ± 8.55 mmol/L in the boldenone group). The Results section has also been systematically checked and residual uses of “co-treatment” and “simultaneous administration” have been replaced where necessary with terminology consistent with the sequential post-treatment design.  

 Comment 5 – Histological image color balance Response: We appreciate the reviewer’s observation. We have carefully re-examined the original histological micrographs. The slight color tint observed is a natural result of the microscope light source and acquisition camera settings rather than any selective digital manipulation. The images presented are the raw, unedited original micrographs that accurately preserve the original histological morphology and pathological findings. No selective editing, filtering, or structural alteration was performed. We have clarified the imaging conditions in the Methods section.  

 Comment 6 – Internal labels and Figure Legend Response: We agree with the reviewer’s suggestion regarding clarity. The figure legends for Figure 1 have been substantially expanded and detailed in the manuscript text to provide comprehensive descriptions of all morphological features and histopathological changes. Furthermore, the legend now explicitly states that the micrographs are representative sections from each experimental group and specifies the exact number of animals analyzed (n = 8 per group).  

 Minor comment 1 – Dose interpretation Response: The manuscript has been revised to clarify that the reported 300 mg/kg/day dose refers to the administered Curcuma longa extract preparation as documented in the experimental protocol, rather than automatically equating the administered dose with a dose of purified curcumin.  

 Minor comment 2 – Figure legends and number of animals Response: The figure legends have been expanded to identify the images as representative sections from the respective experimental groups and to state the number of animals per group (n = 8), consistent with the experimental design described in the manuscript.   Sincerely, Dr. Mohammed Y.I. Al-Hamadani Assistant Professor of Animal Physiology Department of Biology On behalf of all authors

# Reviewer report: Reviewer response for version 1

**DOI:** 10.5256/f1000research.192576.r458730

**Published:** 2026-03-30

**Authors:** Dawei Jiang

**Affiliations:** 1 Huazhong University of Science and Technology, Wuhan, Hubei, China

The manuscript investigates the protective effects of curcumin root extract against kidney injury induced by the anabolic-androgenic steroid boldenone in male Wistar rats. The study addresses a highly relevant toxicological issue, utilizing a comprehensive approach that includes biochemical, oxidative stress, inflammatory, and histopathological markers. However, there are significant discrepancies in the reporting of the experimental design, as well as typographical issues in the data tables that must be addressed to ensure scientific accuracy.

  

1.  There is a major contradiction between the described methodology and the interpretation of the results. In the "Experimental design" section, Group 4 is described as being exposed to boldenone for 4 weeks, followed by 2 weeks of oral curcumin supplementation. This makes it a post-treatment or therapeutic model. However, the Abstract and Results sections refer to this as a "Co-treatment" and a "simultaneous administration". The authors must clarify whether the treatments were given simultaneously or sequentially, and strictly align the terminology throughout the manuscript.
2.  The statistical notations within Tables 1 through 4 are highly irregular and confusing.
3.  In the Discussion, the authors state that high concentrations of anabolic steroids suppress Na/K-ATPase activity, leading to electrolyte imbalances. Since the authors did not actually evaluate Na/K-ATPase expression or activity in their methodology, this claim should be moderated and clearly stated as a hypothesis drawn from existing literature, rather than a direct conclusion of this study.
4.  The "Animal model and housing" and "Experimental design" sections lack details regarding the curcumin root extract. The authors should add a brief clarification regarding the standardization, source, and purity of the curcumin extract used, as this is vital for reproducibility.
5.  The photomicrographs in Figure 1 have a strong yellow tint, which may indicate issues with color balance or white balance during image capture.
6.  The internal textual marks ("G" and "RT") placed directly over the tissue structures in Figure 1 obscure the cellular details. It is recommended to use arrows or asterisks instead, explaining them in the figure legend.

Is the work clearly and accurately presented and does it cite the current literature?

Yes

If applicable, is the statistical analysis and its interpretation appropriate?

Partly

Are all the source data underlying the results available to ensure full reproducibility?

Partly

Is the study design appropriate and is the work technically sound?

Partly

Are the conclusions drawn adequately supported by the results?

Partly

Are sufficient details of methods and analysis provided to allow replication by others?

Partly

Reviewer Expertise:

Drug development for organ injury, in vivo drug delivery, nanomedicine, biomedical engineering, radiopharmaceuticals, molecular imaging

I confirm that I have read this submission and believe that I have an appropriate level of expertise to confirm that it is of an acceptable scientific standard, however I have significant reservations, as outlined above.

# Response

**Published:** 2026-04-02

**Authors:** Mohammed AlHamadani

**Affiliations:** Department of Biology, College, University of Fallujah, Al-Fallujah, Al Anbar Governorate, Iraq

**Response to Reviewer (Dr. Dawei Jiang)**

 We sincerely thank the reviewer for the careful and critical evaluation of our manuscript, as well as for the constructive and insightful comments. We greatly appreciate the reviewer’s focus on experimental clarity, statistical rigor, and reproducibility, which has significantly contributed to improving the overall quality of the manuscript.

 All comments have been carefully considered and addressed. The manuscript has been thoroughly revised, and all corresponding modifications have been incorporated into the revised version.

**Major Comments**

**Comment 1**

*There is a major contradiction between the described methodology and the interpretation of the results (co-treatment vs post-treatment).*

**Response**

 We thank the reviewer for this critical observation.

 We confirm that the experimental design corresponds to a **post-treatment (therapeutic) model**, in which animals were first exposed to boldenone for 4 weeks, followed by 2 weeks of curcumin administration.

 To resolve this inconsistency:

- All instances of “co-treatment” and “simultaneous administration” have been removed.
- The terminology has been standardized throughout the manuscript to **“post-treatment”**.
- The Abstract, Results, and Discussion sections have been revised accordingly.

The following clarification has been consistently incorporated:

*“In the present study, curcumin was administered as a post-treatment intervention following boldenone exposure, rather than as a concurrent co-treatment, to evaluate its therapeutic potential in reversing established renal injury.”*

**This has been revised throughout the manuscript to ensure full consistency.**

**Comment 2**

*The statistical notations in Tables 1–4 are confusing.*

**Response**

 We appreciate this important comment.

 The statistical presentation in Tables 1–4 has been carefully revised to improve clarity and consistency:

- Symbols have been standardized across all tables.
- Table legends have been expanded to clearly define statistical comparisons.
- Formatting inconsistencies have been corrected.

The following unified statement has been added to all table legends:

“Data are expressed as mean ± SE. Statistical comparisons were performed using one-way ANOVA followed by post hoc analysis. Symbols * indicate significant differences compared to the control group, whereas (#) indicate significant differences compared to the boldenone-treated group at *p* < 0.05.”

**These revisions improve readability and eliminate ambiguity in statistical interpretation.**

**Comment 3**

*Na* *⁺* */K* *⁺* *-ATPase was not evaluated but is discussed.*

**Response**

 We fully agree with the reviewer.

 The Discussion has been revised to avoid overinterpretation. The statement regarding Na⁺/K⁺-ATPase has been reformulated as a **literature-supported hypothesis**, rather than a direct finding.

 The following revised sentence has been incorporated:

*“The observed electrolyte imbalance may be partially explained by mechanisms reported in previous studies, including potential impairment of Na* *⁺* */K* *⁺* *-ATPase activity; however, this parameter was not directly assessed in the present study and should therefore be interpreted cautiously.* *”*

**This modification ensures scientific accuracy and appropriate interpretation.**

**Comment 4**

*Lack of details regarding curcumin extract (standardization, source, purity).*

**Response**

 We thank the reviewer for this important comment.

 The Materials and Methods section has been expanded to include clarification regarding the curcumin preparation. The following description has been added:

*“The administered preparation consisted of a curcuminoid-rich Curcuma longa rhizome extract obtained in powder form and freshly suspended in distilled water prior to administration. The selected dose (300 mg/kg/day) was chosen based on previously published nephroprotection studies. The preparation represents a standardized extract commonly used in experimental pharmacological studies to ensure reproducibility of biological activity.”*

**This addition improves reproducibility and methodological transparency.**

**Comment 5**

*Photomicrographs have a strong yellow tint.*

**Response**

 We appreciate this observation.

 The slightly warm (yellowish) tone observed in the histological sections is consistent with normal variability associated with H&E staining and imaging conditions, rather than an artifact affecting data quality or interpretation.

 Minor adjustments to brightness and contrast were applied to enhance overall clarity while preserving the original histological features. No alterations were made that could affect the scientific interpretation of tissue morphology.

 We confirm that the images accurately represent the observed histopathological findings and are suitable for scientific evaluation.

 This has been carefully refined in the revised figure.

**Comment 6**

*Internal labels (G and RT) obscure tissue structures.*

**Response**

 We agree with the reviewer.

- Internal labels (“G” and “RT”) have been removed from the images.
- Tissue structures are now described in detail within the figure legend.
- Where necessary, descriptive labeling has been shifted to the legend rather than the image.

**This has been revised in Figure 1 and its legend.**

**General Evaluation Questions**

**Is the work clearly and accurately presented, and does it cite current literature?**

 Yes. The manuscript has been further improved and updated.

**Is the study design appropriate and technically sound?**

 Yes. The experimental design is appropriate and has been clarified.

**Are sufficient methodological details provided?**

 Yes. Additional clarification regarding curcumin extract has been added.

**Is the statistical analysis appropriate?**

 Yes. Statistical presentation has been standardized and clarified.

**Are all data available?**

 Yes. All data are publicly available in the Zenodo repository.

**Are conclusions supported by the results?**

 Yes. The conclusions have been revised to align strictly with the findings.

 We sincerely thank the reviewer again for the valuable comments, which have significantly strengthened the manuscript.

# Reviewer report: Reviewer response for version 1

**DOI:** 10.5256/f1000research.192576.r458721

**Published:** 2026-03-23

**Authors:** Daniela Joyce Trujillo

**Affiliations:** 1 Instituto Potosino de Investigación Científica y Tecnológica (DMA-IPICYT), San Luis, Mexico

The manuscript evaluates the potential protective effect of curcumin root extract against boldenone-induced kidney injury in male rats through biochemical, oxidative stress, inflammatory, and histological parameters. The topic is relevant, and the results are, in general, consistent with the proposed objective. However, some aspects of the introduction, data presentation, image quality, and discussion should be improved before indexing. Therefore, major revisions are required.

**Major points**

1. In the Introduction, the authors should explain in more detail the mechanisms by which boldenone or anabolic steroids may induce nephrotoxicity. In addition, it will strengthen the rationale if they mention whether systemic biomarkers of this toxicity have been previously reported.
2. The statistical presentation in tables is confusing. The use of the same symbols for all columns makes it difficult to identify the exact comparisons. The statistical notation should be clarified and better explained in each table legend.
3. The quality of the microphotographs in Figure 1 should be improved. The background appears too yellow, and the authors should clarify whether the contrast or color balance was excessively modified. It is also suggested to remove the internal marks “G” and “RT” from the images.
4. In the Results section, it would have been valuable if the authors had evaluated Na⁺/K⁺-ATPase expression, especially because this mechanism is later mentioned in the Discussion. If this was not evaluated, the discussion should be moderated accordingly.
5. In the Discussion, the authors should better justify why studying compounds that may counteract the harmful effects of steroid abuse is scientifically relevant. This would strengthen the translational importance of the study.
6. The manuscript should more clearly distinguish whether curcumin was used as a co-treatment or as a post-treatment strategy, since this affects the interpretation of the protective effect.

**Minor points**

1. The manuscript would benefit from improvement in English writing and sentence structure in several sections.
2. The authors should revise the consistency of terms and descriptions across the Results and Discussion sections.
3. It is suggested to improve the explanation of the histological findings in the figure legends.
4. The conclusions should be slightly moderated and limited to the experimental model.

Is the work clearly and accurately presented and does it cite the current literature?

Partly

If applicable, is the statistical analysis and its interpretation appropriate?

Partly

Are all the source data underlying the results available to ensure full reproducibility?

Partly

Is the study design appropriate and is the work technically sound?

Partly

Are the conclusions drawn adequately supported by the results?

Partly

Are sufficient details of methods and analysis provided to allow replication by others?

Partly

Reviewer Expertise:

kidney phisiology and metabolic diseases

I confirm that I have read this submission and believe that I have an appropriate level of expertise to confirm that it is of an acceptable scientific standard, however I have significant reservations, as outlined above.

# Response

**Published:** 2026-03-23

**Authors:** Mohammed AlHamadani

**Affiliations:** Department of Biology, College, University of Fallujah, Al-Fallujah, Al Anbar Governorate, Iraq

**Response to Reviewer**

 We sincerely thank the reviewer for the thorough and constructive evaluation of our manuscript. We greatly appreciate the insightful comments, which have significantly improved the clarity, rigor, and scientific value of the work. All comments have been carefully addressed, and the manuscript has been revised accordingly. The corresponding modifications have been incorporated into the revised version of the manuscript.

**Major Comments**

**Comment 1**

*In the Introduction, the authors should explain in more detail the mechanisms by which boldenone or anabolic steroids may induce nephrotoxicity. In addition, it will strengthen the rationale if they mention whether systemic biomarkers of this toxicity have been previously reported.*

**Response**

 We thank the reviewer for this important suggestion. The Introduction section has been expanded to provide a clearer mechanistic background and to highlight relevant systemic biomarkers. The following paragraph has been added to the manuscript:

*“Anabolic-androgenic steroids such as boldenone are known to induce nephrotoxicity through multiple interconnected mechanisms, including excessive generation of reactive oxygen species (ROS), lipid peroxidation, and activation of pro-inflammatory pathways such as TNF-α signaling. In addition, these compounds may disrupt renal electrolyte homeostasis through interference with membrane transport systems, including Na* *⁺/K* *⁺-ATPase activity, leading to ionic imbalance and cellular dysfunction. Structural alterations such as glomerular hyperfiltration and tubular hypertrophy further contribute to progressive renal injury. Clinically, these pathological changes are reflected in elevated serum creatinine and urea levels, along with electrolyte disturbances, which are widely recognized as systemic biomarkers of steroid-induced nephrotoxicity.”*

**This has been revised in the Introduction section of the manuscript.**

**Comment 2**

*The statistical presentation in tables is confusing.*

**Response**

 We appreciate this observation. The statistical presentation in Tables 1–4 has been revised to improve clarity. The following clarification has been added to all table legends:

*“Data are expressed as mean ± SE. Statistical comparisons were performed using one-way ANOVA followed by post hoc analysis. Symbols (*) indicate significant differences compared to the control group, whereas (#) indicate significant differences compared to the boldenone-treated group at p < 0.05.”*

**This has been revised in all table legends.**

**Comment 3**

*Improve the quality of microphotographs and remove G and RT.*

**Response**

 We thank the reviewer for this valuable comment. Figure 1 has been improved, and internal labels have been removed. The figure legend has been revised as follows:

*“Figure 1. Photomicrographs of H&E-stained kidney sections. Control and curcumin-treated groups show normal renal architecture with intact glomeruli and well-organized renal tubules. In contrast, boldenone-treated rats exhibit marked tubular degeneration, cellular necrosis, and structural disorganization. Post-treatment with curcumin markedly attenuated these alterations, with partial restoration of normal renal histology and only mild residual changes.”*

**This has been revised in Figure 1 and its legend.**

**Comment 4**

*Na* *⁺/K* *⁺-ATPase was not evaluated but is discussed.*

**Response**

 We agree with the reviewer. The Discussion has been revised to avoid overinterpretation. The following modified sentence has been incorporated:

*“The observed electrolyte imbalance may be partially explained by mechanisms reported in previous studies, including potential impairment of Na* *⁺/K* *⁺-ATPase activity; however, this parameter was not directly assessed in the present study and should therefore be interpreted cautiously.* *”*

**This has been revised in the Discussion section.**

**Comment 5**

*Justify relevance of studying protective compounds against steroid abuse.*

**Response**

 We appreciate this important point. The following paragraph has been added to the Discussion:

*“Given the increasing global misuse of anabolic-androgenic steroids and the associated risk of renal and systemic toxicity, the identification of safe and effective protective agents is of considerable clinical and public health importance. Natural compounds such as curcumin, with well-documented antioxidant and anti-inflammatory properties, may offer a promising adjunct strategy to mitigate steroid-induced organ damage and reduce long-term health complications.”*

**This has been revised in the Discussion section.**

**Comment 6**

*Clarify whether curcumin is a co-treatment or post-treatment.*

**Response**

 We thank the reviewer for this critical clarification. The manuscript has been revised to clearly define the treatment strategy. The following clarification has been added:

*“In the present study, curcumin was administered as a post-treatment intervention following boldenone exposure, rather than as a concurrent co-treatment, in order to evaluate its therapeutic potential in reversing established renal injury.”*

**This clarification has been consistently applied in the Materials and Methods, Results, and Discussion sections.**

**Minor Comments**

**Comment 7 – Language improvement**

**Response:** The manuscript has been carefully revised for grammar and clarity.

**Comment 8 – Consistency**

**Response:** Terminology and descriptions have been standardized across all sections.

**Comment 9 – Histological explanation**

**Response:** Figure legends have been expanded to improve clarity.

**Comment 10 – Conclusion moderation**

**Response**

 The Conclusion has been revised as follows:

*“In conclusion, boldenone administration induces significant renal dysfunction associated with oxidative stress, inflammation, and histopathological alterations in male rats. Curcumin root extract demonstrated a protective effect by ameliorating these changes, likely through its antioxidant and anti-inflammatory properties. However, these findings are limited to an experimental animal model, and further studies are required to elucidate the underlying molecular mechanisms and to confirm their relevance in clinical settings.”*

**This has been revised in the Conclusion section.**
